# Supplementary material for: Estimation of nonclassical independent Gaussian processes by classical interferometry
Source: Sci Rep. 2017 Jan 4;7:39641. doi: 10.1038/srep39641 (PMC5209653; doi:10.1038/srep39641)
Supplement: Supplementary Information [file srep39641-s1.pdf]

Supplementary for "Estimation of nonclassical independent Gaussian process by classical interferometry"

László Ruppert[1,*],Radim Filip[1,+]

Department of Optics, Palacky University, 17. listopadu 12, 771 46 Olomouc, Czech Republic
[*] ruppert@optics.upol.cz
[+]filip@optics.upol.cz

1 Approximation of Fisher information and mean squared error

The estimators of the unknown parameters are non-linear, therefore their quality (FI or MSE) cannot be calculated analytically. In the following we will describe an approximation for the estimator of the squeezing parameter q for a thermal source and passive optical elements (beam splitters). For different setups (i.e., using a displaced thermal source, active optical elements or different estimators) the calculations can be performed similarly, but we refrain from it here because the conclusion would be the same.

Let us remind ourselves that the estimator of the squeezing magnitude is

$$\hat{q} = \frac{c + \sqrt{c^2 - 4}}{2}, \tag{1}$$

where

$$c = 2\frac{\sqrt{\langle i_- \rangle^2_{\varphi=\pi/2} + \langle i_- \rangle^2_{\varphi=0}}}{\sqrt{\mu(1-\mu)}(V-1)}. \tag{2}$$

Mean squared error

The MSE cannot be calculated analytically since $\langle i_- \rangle$ is already complicated enough so that its likelihood function can be problematic to give. Moreover, we need to perform non-linear transformations to obtain the value of $\hat{q}$, so to calculate $MSE(\hat{q})$ we need to apply some approximation. In the main text we used numerical simulation and empirical MSE, which has the advantage that with a large enough iteration number (M) we can get arbitrarily close to the real MSE. Here we will approximate the distribution with a normal distribution with the same mean and variance. As we will see it results in a good approximation for large numbers of measurements (N) and since it is a closed formula it can be calculated instantaneously. However, it can be imprecise for small values of N.

The properties of c can be calculated straightforwardly:

$$m := \mathbb{E}(c) = q + \frac{1}{q} \tag{3}$$

and

$$\sigma^2 := \mathrm{Var}\,(c) = \frac{1}{N}\left(q^2 + \frac{1}{q^2}\right)\left(2 + \frac{V}{(1-\mu)\mu(V-1)^2}\right) \tag{4}$$

The value of c^2 can be calculated using this, since if we assume c is normally distributed, then c^2 will be non-central χ^2 distributed. While c^2-4 is only displaced by the mean of -4, so we obtain

$$\mathbb{E}(c^2-4) = m^2+\sigma^2-4 \tag{5}$$

and

$$\mathrm{Var}\,(c^2-4) = 2\sigma^2(2m^2+\sigma^2). \tag{6}$$

Once again c^2-4 is not normally distributed, but it can be approximated (for a certain regime) with a normal distribution. Then its mean can be calculated using Tricomi's confluent hypergeometric U-function:

$$\mathbb{E}\sqrt{c^2-4} = \frac{1-i}{2^{1/4}}\cdot \mathrm{Var}\,^{1/4}(c^2-4)\cdot U\biggl(-\frac{1}{4},\frac{1}{2},-\frac{\mathbb{E}^2(c^2-4)}{2\mathrm{Var}\,(c^2-4)}\biggr), \tag{7}$$

and the variance is

$$\mathrm{Var}\,\sqrt{c^2-4} = \mathbb{E}(c^2-4)-\mathbb{E}^2\sqrt{c^2-4}. \tag{8}$$

Using the above it is already easy to obtain the properties of $\hat{q}$:

$$\mathbb{E}(\hat{q}) = \frac{\mathbb{E}(c)+\mathbb{E}\sqrt{c^2-4}}{2} \tag{9}$$

and

$$\mathrm{Var}\,(\hat{q}) = \frac{\mathrm{Var}\,(c)+\mathrm{Var}\,\sqrt{c^2-4}}{2} \tag{10}$$

From (9) it is visible that $\hat{q}$ is not an unbiased estimator, numerically one can check that it is still asymptotically unbiased. If the bias is small and the normal approximation is valid, then (10) will be close to the actual MSE of $\hat{q}$.

Fisher information

The situation is similar in the case of Fisher information. Let us derive the maximally available information on q from $\langle i_-\rangle$. For that we approximate $\langle i_-\rangle$ with a normal distribution. Its mean is

$$\mathbb{E}(\langle i_-\rangle) = \frac{V-1}{2}\sqrt{\mu(1-\mu)}\biggl(q+\frac{1}{q}\biggr)\cos(\Phi-\varphi). \tag{11}$$

The last term is a constant multiplier, so let us use $\varphi=\Phi$ to obtain the maximal FI. In this case the variance will be

$$\mathrm{Var}\,(\langle i_-\rangle) = \frac{1}{4N}\biggl(q^2+\frac{1}{q^2}\biggr)\biggl(2(1-T)T(V-1)^2+V\biggr). \tag{12}$$

Let $f(x,q)$ be the likelihood function of a normal distribution with the same mean and variance. Then the Fisher information can be calculated as

$$I_{normal} = \int\biggl(\frac{\partial\log f(x,q)}{\partial q}\biggr)f(x,q)dx. \tag{13}$$

The derivative of the likelihood function can be expressed explicitly and can be rewritten in the following form:

$$I_{normal} = \int p_4(x)e^{-a(x-b)^2}dx, \tag{14}$$

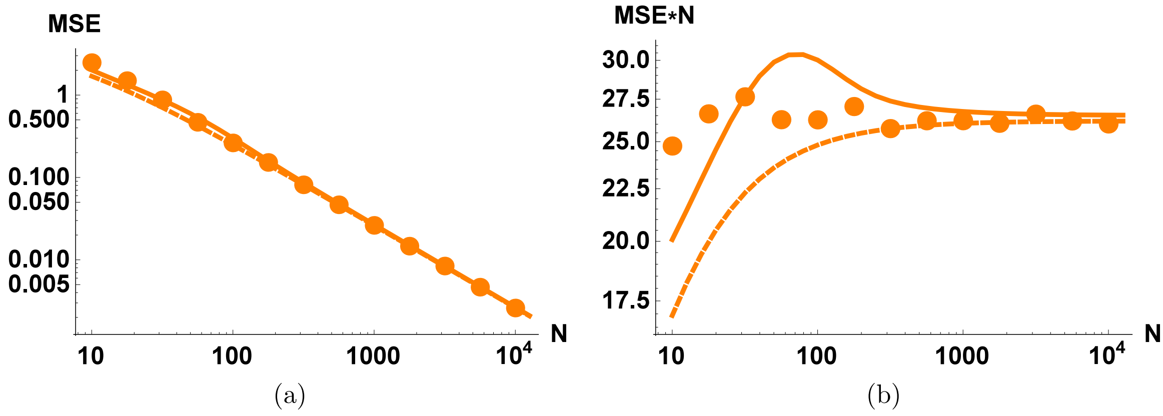

Figure 1: MSE (a) and MSE$\cdot N$ (b) of the estimation of squeezing parameter q with different approximations: dots correspond to the numerical simulation, solid lines to the approximated variance, dashed lines come from Cramér-Rao inequality using the approximated Fisher information. We have parameters $R = 5$, $D = 0$, $T = 0.2$ and $q = 3$.

where $p_4(x)$ is a fourth order polynomial, a and b are functions of the parameters. Since $\int_{-\infty}^{\infty} x^k e^{-a(x-b)^2} dx$ can be expressed analytically for every value of k, in principle we can calculate the Fisher information analytically, however, its formula will be too extensive to include here (nevertheless, for a computer it is not a problem).

The Fisher information is related to the variance through the Cramér-Rao inequality:

$$\mathrm{Var}\left(\hat{q}\right) \geq I^{-1}, \tag{15}$$

that is, the inverse of the Fisher information is a lower bound for the variance, and so also for the MSE. However, it is unclear whether we can get close to this bound or not. Another issue is that we have only an approximation of the Fisher information; where the normal approximation does not hold, we do not know how the actual Fisher information and I_{normal} are related.

Numerical results

The plots of a particular case can be seen in Fig. 1. In the left subfigure we can see that the three methods produce very similar errors. The MSE is of order $1/N$, so we plotted the normalized version too (right subfigure). Here, we can see better that for large values of N the values from the numerical simulation almost coincide with the approximation of the variance and the Fisher information. This is expected since according to the central limit theorem the given distributions indeed converge to a normal distribution. So for large values of N the approximation will be really good. On the other hand, below $N = 1000$ the 3 curves separate. The magnitude of the differences in the current settings goes up to 50%, which is in some situations significant. So we used the numerical simulation even though its calculation is the slowest and does not give smooth curves, just because it is working in all regimes, while the other approximations can give us systematically wrong results.

2 The effect of loss and noise

Channel-type errors

First, we will investigate the effect of errors by applying a channel between the preparation and the measurement phases to both the signal and the reference mode. The channel is modeled by a beam-splitter coupled

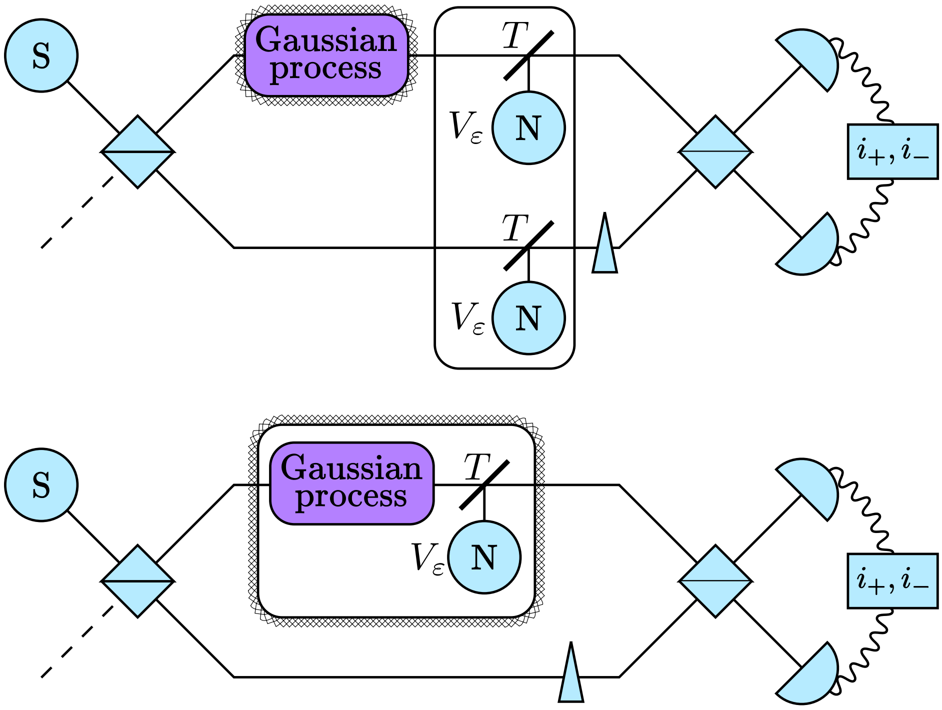

Figure 2: Schematic setup for channel-type errors (upper subfigure) and for process-type errors (lower subfigure).

to a thermal source (Fig. 2 upper subfigure). The transmittance of the beam splitter (T) defines the loss of the channel, the variance of the auxiliary mode (V_ε) defines the noise of the channel.

If we use the estimators defined in the main part the estimation of the parameters can be biased. Indeed, by increasing the number of measurements the estimation cannot converge to the real values of the parameters of the unknown process (Fig. 3, dotted lines). Therefore the estimators obtained in the noiseless case have limited applicability in a realistic setting; we should take into account the effect of the channel as well (once again, we will discuss in detail only the BS case, the OPA case can be handled using the same steps with similar calculations).

If we include also the noise and the loss in our calculations, we have for the expected difference in the photon numbers:

$$\langle i_- \rangle = T \cdot \frac{V-1}{2} \sqrt{\mu(1-\mu)} \left(q + \frac{1}{q} \right) \cos(\Phi - \varphi) \tag{16}$$

and for the sum of the photon numbers:

$$\langle i_+ \rangle = T \cdot \frac{\left(q^2 + \frac{1}{q^2} \right) V_S + 2V_R + d^2}{4} + V_\varepsilon (1-T) - 1. \tag{17}$$

The simplest way to estimate the parameters of the channel is to calibrate the setup prior to the actual measurements. In the first step, we do not modify the signal (i.e., $\rho^* = \rho$), and we let the unaltered signal and the reference pass through the channel.

In mathematical terms that means that we should substitute into (16)-(17) the values $q = 1, d = \Phi = \varphi = 0$:

$$\langle i_- \rangle_{\text{calibr.}} = T \cdot (V-1) \sqrt{\mu(1-\mu)} \tag{18}$$

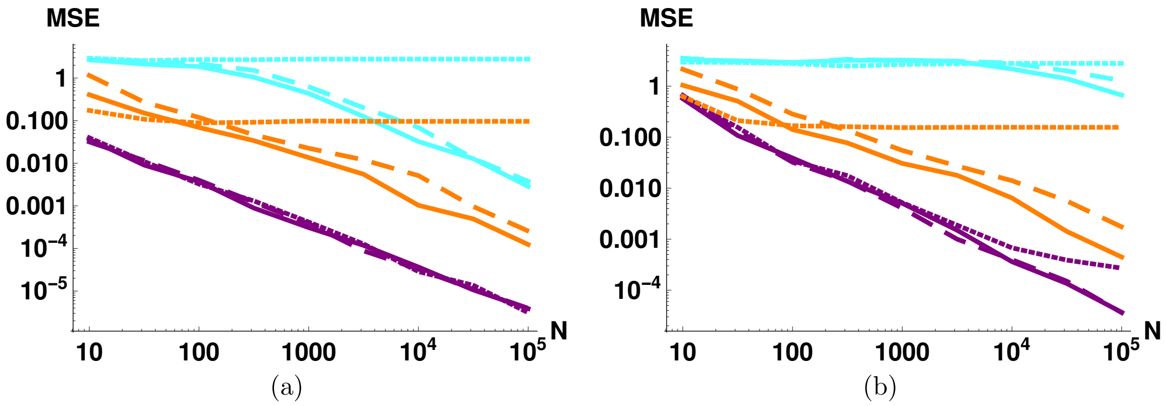

Figure 3: MSE of the process estimation with channel-type error as a function of N (a) for BS and (b) for OPA. Cyan (light) lines correspond to the estimation of the displacement d, orange (medium) to the squeezing q and purple (dark) to the phase-shift Φ. We plotted the cases when we know the noise a priori (solid lines), we estimate the noise (dashed lines), we assume that the channel is ideal (dotted lines). We have parameters $V = 75$, $q = 1.23$, $\Phi = 0.63$, $d = 1.67$, $T = 0.9$, $V_\varepsilon = 1.1$, for (a) $\mu = 0.3$ and for (b) $r = 0.5$.

and

$$\langle i_+ \rangle_{\text{calibr.}} = T \cdot \frac{V}{2} + V_\varepsilon(1-T) - 1; \tag{19}$$

from which we can estimate the value of the loss

$$\hat{T} = \frac{\langle i_- \rangle_{\text{calibr.}}}{(V-1)\sqrt{\mu(1-\mu)}}. \tag{20}$$

and the noise

$$\hat{V}_\varepsilon = \frac{\langle i_+ \rangle_{\text{calibr.}} + 1 - \hat{T}(V+1)/2}{1-\hat{T}}. \tag{21}$$

Using these we can get unbiased estimates of the parameters T and V_ε.

Now by knowing the channel parameters, in the second step we can solve Eq. (16)-(17) similarly as in the ideal case, obtaining estimators for the unknown parameters Φ, q and d (Fig. 3, dashed lines). We can see that these estimators are almost as efficient as if we knew the channel parameters T and V_ε precisely (Fig. 3, solid lines). That is, by using a calibration round channel-type noises can be handled exceptionally well.

Process-type errors

The second naturally arising error can appear during the implementation of the Gaussian process. This error is also modeled by a beam-splitter coupled to a thermal source (Fig. 2 upper subfigure), with a loss of T and a noise of V_ε. The difference is that in this case the reference is unaltered, and more importantly, we cannot use a calibration round without applying the process since the error is only present if the unknown Gaussian process is present, too.

If we use the "naive" estimators defined for the ideal case, the estimation of the parameters will be biased again (Fig. 4, dotted lines). Therefore we have to estimate the errors, but since we cannot estimate the errors in an independent calibration round, we can only do so by repeating the experiment twice using sources with different strengths (i.e., having V_1 and V_2 instead of a single V).

Now for process-type errors instead of Eq. (16) we have

$$\langle i_- \rangle_V = \sqrt{T}\left(q + \frac{1}{q}\right) \cdot \frac{V-1}{2}\sqrt{\mu(1-\mu)}\cos(\Phi - \varphi) \tag{22}$$

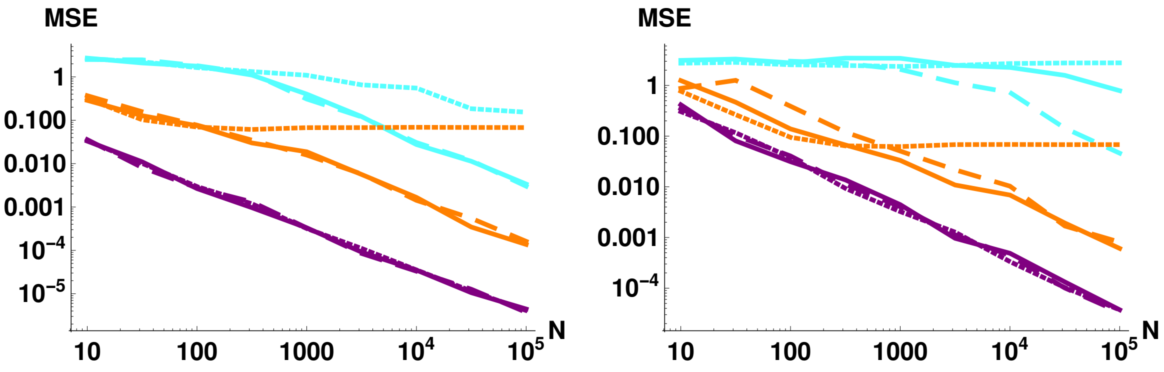

(a) (b)

Figure 4: MSE of the process estimation with process-type error as a function of N (a) for BS and (b) for OPA. Cyan (light) lines correspond to the estimation of the displacement d, orange (medium) to the squeezing q and purple (dark) to the phase-shift Φ. We plotted the cases when we know the noise a priori (solid lines), we estimate the noise (dashed lines), we assume that the channel is ideal (dotted lines). We have parameters $V_1 = 75$, $V_2 = 300$, $q = 1.23$, $\Phi = 0.63$, $d = 1.67$, $T = 0.9$, $V_\varepsilon = 1.1$, for (a) $\mu = 0.3$ and for (b) $r = 0.5$.

and instead of Eq. (17) we obtain

$$\langle i_+\rangle_V = T\cdot\frac{\left(q^2+\frac{1}{q^2}\right)V_S + d^2}{4} + \frac{V_R}{2} + \frac{V_\varepsilon(1-T)}{2} - 1. \tag{23}$$

We can estimate Φ the usual way from (22). Using two different values of V in (23) we gain an alternative equation:

$$\langle i_+\rangle_{V_2} - \langle i_+\rangle_{V_1} = T\left(q^2+\frac{1}{q^2}\right)\cdot\frac{\mu\Delta V}{4} + \frac{(1-\mu)\Delta V}{2}, \tag{24}$$

with $\Delta V = V_2 - V_1$, which combined with (22) provides sufficient information to estimate the parameters T and q beside Φ. However, the effects of the displacement d and the noise V_ε cannot be distinguished. Neither appears in the interference, they only result in an additional increase in the mean number of photons (i.e., both are seemingly just noise), thus to distinguish them we should use a phase-sensitive measurement. However, if the noise is small and we assume that all of the additional energy is coming from the displacement, we can still have a reasonable estimation precision (Fig. 4, dashed lines). The efficiency of these estimators is almost as good as if we knew the error parameter precisely (Fig. 4, solid lines).
